# Social disparities in patient safety in primary care: a systematic review

**DOI:** 10.1186/s12939-018-0828-7

**Published:** 2018-08-07

**Authors:** Carlotta Piccardi, Jens Detollenaere, Pierre Vanden Bussche, Sara Willems

**Affiliations:** 1 Department of Family Medicine and Primary Health Care, Ghent University, Campus UZ, 6K3, C Heymanslaan 10, 9000 Ghent, Belgium; 2 European Association for Quality and Patient Safey in Primary Care (WONCA Europe Network), Copenhagen, Denmark; 3 Centre for the Social Study of Migration and Refugees (CESSMIR), Ghent University, Ghent, Belgium

**Keywords:** Patient safety, Primary care, Inequity, Education, Income, Ethnicity, Gender, High-income countries

## Abstract

**Background:**

Patient safety is a quality indicator for primary care and it should be based on individual needs, and not differ among different social groups. Nevertheless, the attention on social disparities in patient safety has been mainly directed towards the hospital care, often overlooking the primary care setting. Therefore, this paper aims to synthesise social disparities in patient safety in the primary care setting.

**Methods:**

The Databases PubMed and Web of Science were searched for relevant studies published between January 1st 2006 and January 31st 2017**.** Papers investigating racial, gender and socioeconomic disparities in regards to administrative errors, diagnostic errors, medication errors and transition of care errors in primary care were included. No distinction in terms of participants’ age was made.

**Results:**

Women and black patients are more likely to experience patient safety events in primary care, although it depends on the type of disease, treatment, and healthcare service. The available literature largely describes gender and ethnic disparities in the different patient safety domains whilst income and educational level are studied to a lesser extent.

**Conclusions:**

The results of this systematic review suggest that vulnerable social groups are likely to experience adverse patient safety events in primary care. Enhancing family doctors’ awareness of these inequities is a necessary first step to tackle them and improve patient safety for all patients. Future research should focus on social disparities in patient safety using socioeconomic indicators, such as income and education.

## Introduction

Since the Institute of Medicine (IOM) released its seminal report “To Err is Human” in 1999, patient safety caught the public’s attention as few other healthcare policies have done before [1]. Patient safety is the absence of preventable harm to a patient such as results of a wrong diagnosis, clinical procedure, side-effects of drugs, or system errors during the process of healthcare and therefore it is the minimum prerequisite for high-quality care [2]. European data show that the issue of patient safety is on-going and that, for example, in the United Kingdom between 5 and 80 safety incidents occur per 100,000 primary care consultations, which translates to between 370 and 600 incidents per day [3]. Considering these numbers it is understandable how patient safety is generally seen as one of the most pressing healthcare challenges. Paradoxically, although most of the care is provided in the primary care setting, the attention on patient safety has been largely focussed on the specialist care setting. This inattention to patient safety in primary care might be explained by the fact that primary care is sometimes perceived as less risky than secondary care [4]. For this reason, attention towards patient safety was renewed in 2016 by the World Health Organisation (WHO) with its “Technical Series on Safer Primary Care” aiming at raising awareness about the underlying causes of safety incidents and consequences of unsafe primary care [5]. Patient safety events resulting from the happenstance of mistakes and errors should not occur systematically across racial, ethnic, or socioeconomic subgroups [6]. To the best of our knowledge, social disparities in patient safety in the primary care setting are not yet explored in a comprehensive way, that is accounting for multiple individual and socioeconomic determinants simultaneously. Thus, this study aims to synthesise the existing literature of patient safety in primary care categorised under the most relevant domains of the WHO framework, namely administrative procedure errors, diagnostic and medication errors and transition of care errors and to explore whether these events vary according to gender, ethnicity, income, and education.

## Methods

### Search strategy

A literature search was conducted using two databases: PubMed and Web of Science. As the topic of patient safety is susceptible to changes over time, the search was limited to publications published between January 1st 2006 and January 31st 2017.

The lack of funds for translation of publications made it necessary to restrict this systematic review to publications published in languages mastered by the researchers namely English, French, Dutch and Italian. The search terms were based on the patient-safety domains according to the WHO-framework [5] and consequently discussed with patient safety experts in order to increase the quality of the search strategy. The search strategy is presented in Table 1*.* During the screening stage, no distinction was made in terms of research design and of population’s age, including studies on adults, adolescents and children. Only studies on primary care, ethnicity, gender, income, education and that were carried out in high-income countries (World Bank Classification) were included.

**Table 1:** Search strategy

"Primary care OR Family Practice OR Family Medicine" [all fields]	AND	"Patient safety"^a^	AND	"Inequalit* OR inequit* OR disparit* OR Socioeconomic disparit* OR Socioeconomic difference* OR Socioeconomic status OR Socioeconomic factor* OR Socioeconomic level OR Social class OR Social position OR Social hierarchy OR Gender OR Ethnicity OR Educational achievement OR Educational attainment"
		"Adverse events"^a^		
		"Adverse effects"^a^		
		"Safety management"^a^		
		"Medication error"^b^		
		"Administrative errors"^c^		
		"Organizational errors"^c^		
		"Diagnostic errors"^d^		
		"Over-diagnosis"^d^		
		"Under-diagnosis"^d^		
		"Missed diagnosis"^d^		
		"Medical error"^d^		
		"Transitional care"^e^		

^a^search terms for patient safety

^b^search terms for medication errors

^c^search terms for administrative errors

^d^search terms for diagnostic errors

^e^search terms for transition of care errors

Results of the search strategy were uploaded in COVIDENCE, a software developed by Cochrane Library to facilitate and improve the collaboration among reviewers.

### Study selection and inclusion

**Fig. 1:**
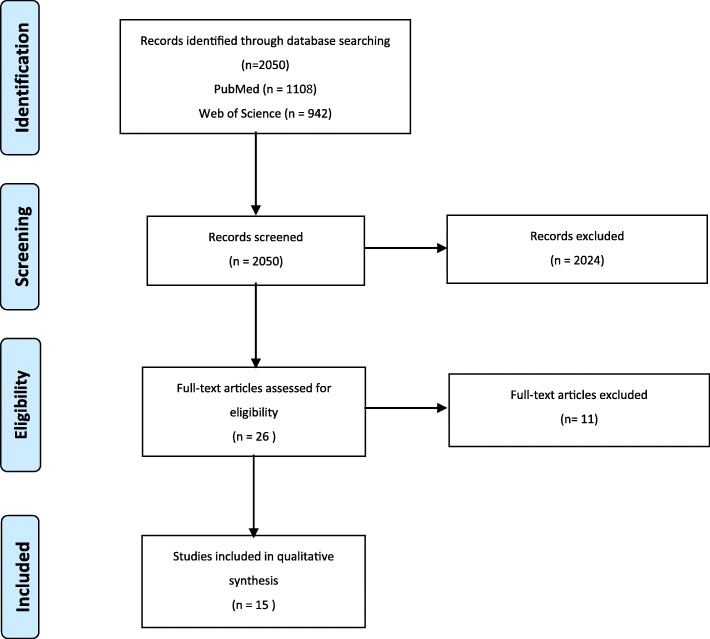
PRISMA diagram for study selection

Figure 1 provides an overview of the study selection. A total of 2050 studies were retrieved. At the first screening round, all titles and abstracts were screened independently by two reviewers (JD and CP) and 2024 articles were excluded. In the second round, the full-texts of the 26 remaining studies were reviewed independently by the two researchers (JD and CP). During this round, 11 articles were excluded. Disagreement was automatically recorded in COVIDENCE at each screening stage and discussed until consensus was reached. Eventually, a total of 15 studies were included for analysis.

For each included study, a data-extraction form and a quality assessment were completed by two independent researchers (JD and CP). The standardised Quality Assessment Tool for Observational Cohort and Cross-Sectional Studies was used [7]. For the included systematic review, the Critical Appraisal Skills Programme (CASP) was used. Using these instruments, the included publications were rated good, fair or poor.

To ensure that the two reviewers were collecting the same information from each study, the use of the Quality Assessment Tool was first piloted and tested. Hereto the researchers independently used the tool in the assessment of five papers and then discussed the discrepancies in their analysis. They analyzed whether these discrepancies occurred due to a different interpretation of the items in the Quality Assessment Tool and came to consensus on the points where their interpretation was different. Also a method for dispute resolution was discussed a priori.

Given the heterogeneity of study population, study design, interventions and outcomes, it is not possible to conduct a meta-analysis. Information extracted from the included studies can be consulted in Table 2 . This table provides information regarding first author, publication date, country, study design, study population, outcome measure and relevant study outcomes.

**Table 2:** Description of the included studies

N	Citation	Location	Outcome of interest	Patient safety domain	Study design	Major findings	Quality of the study
1	Maserejian et al. (2009) [22]	USA	Gender disparities in physicians’ diagnosis of coronary heart disease	Diagnostic error	Factorial experiment	Gender: diagnosis of coronary heart disease is significantly dependent on patient’s gender: women are less likely to be diagnosed with coronary heart disease; despite identical symptoms. Ethnicity: not associated with the diagnosis of coronary heart disease. Income: high income women more likely to receive a mental health diagnosis instead of coronary heart disease diagnosis. Education: not studied.	Fair
2	Hansen et al. (2008) [12]	DK	Socioeconomic patients characteristics influencing delay in cancer diagnosis	Transition of care/ diagnostic error	Cross-sectional Study	Gender: doctor and system delays: male cancer patients experience longer delays than female cancer patients. Ethnicity: not studied. Income: high income associated with shorter doctor and systems delays and longer patient delays. Education: well educated males and well educated patients in general, experience shorter doctor delays.	Good
3	Henning et al. (2013) [16]	AU & IT	Gender differences in referral patterns for bladder cancer	Diagnostic error	Cross-sectional Study	Gender: men are 65% more likely to be referred to a specialist at the first episode of haematuria compared to women. Ethnicity: not studied. Income: not studied. Education: not studied.	Fair
4	Kistler et al. (2010) [18]	USA	Patient characteristics influencing the perceptions of mistakes in ambulatory care	Administrative error	Cross-sectional Study	Gender: gender not associated with perception of mistakes. Ethnicity: no association between ethnicity and perception of mistakes. Income: not studied. Education: not studied.	Fair
5	Maeng et al. (2012) [21]	USA	Perception of care coordination problems	Administrative error	Cross-sectional Study	Gender: not studied. Ethnicity: ethnicity not associated with self-reported care coordination problems. Income: income not associated with self-reported care coordination problem. Education: not studied.	Fair
6	McKinlay et al. (2012) [13]	USA	Racial disparities in diabetes mellitus diagnosis	Diagnostic error	Mixed methods: survey, factorial experiment	Gender: not studied. Ethnicity: White patients, with the same symptoms as black patients and Hispanics, underdiagnosed with diabetes mellitus type 2. Income: Undiagnosed signs and symptoms of diabetes mellitus type 2 patterned by income and education. Education: Undiagnosed signs and symptoms of diabetes mellitus type 2 patterned by income and education.	Good
7	Eva et al. (2010) [9]	USA	Factors related to physicians’ changing their minds about a diagnosis	Diagnostic error	Factorial experiment	Gender: gender is no significant predictor of change of diagnosis. Ethnicity: ethnicity is no significant predictor of change of diagnosis. Income: income no significant predictor of change of diagnosis. Education: education no significant predictor of change of diagnosis.	Good
8	Cooper et al. (2016) [15]	GBR& IRL	Socioeconomic patients’ characteristics influencing potentially inappropriate prescriptions	Medication error	Cross-sectional Study	Gender: women have increased likelihood of potentially inappropriate prescriptions compared to men. Ethnicity: not studied. Income: low income patients have increased risk of potentially inappropriate prescriptions compared to their wealthier counterparts. Education: not studied.	Fair
9	Becker et al. (2011) [8]	USA	Racial disparities in opioid risk reduction strategies	Medication error	Retrospective Cohort Study	Gender: not studied. Ethnicity: black patients are more likely to receive opioid risk reduction strategy compared to white patients. Income: not studied. Education: not studied.	Good
10	Ladapo et al. (2014) [19]	USA	Patients’ characteristics influencing physicians’ decision making for cardiac stress testing use	Transition of care	Cross-sectional Study	Gender: women increased likelihood of undergoing or being referred for cardiac testing. Ethnicity: No association between black race and Hispanic ethnicity and lower likelihood of receiving cardiac stress test compared to whites. Income: not studied. Education: not studied.	Fair
11	Lukakcho & Olfson (2012)	USA	Racial difference of depression diagnosis during first primary care visit	Diagnostic error	Cross-sectional study	Gender: not studied. Ethnicity: African American patients more likely to be underdiagnosed with depression during the first GP visit compared to white patients. Income: not studied. Education: not studied.	Fair
12	Hickner et al. (2007)	USA	Predictors of adverse events due to testing errors.	Administrative error	Cross-sectional Study	Gender: not studied. Ethnicity: minority patients have higher odds of experiencing adverse consequences due to testing errors compared to white and non-Hispanic patients. Income: not studied. Education: not studied.	Fair
13	Schröder et al. (2016) [14]	NZL, ESP, SWE, ITA, BEL, DNK, DEU, ISR & GBR	Gender differences in antibiotic prescription	Medication error	Systematic review	Gender: Women are 27% more likely than men to receive antibiotic prescription; The amount of antibiotics prescribed to women is 36% higher than that prescribed to men in the 16–34 years age group and 40% higher in the 35–54 years age group. In particular, the amount of cephalosporins and macrolides prescribed to women is 44 and 32% higher, respectively, than those prescribed to men. Ethnicity: not studied. Income: not studied. Education: not studied.	Good
14	Green et al. (2013) [11]	GBR	Factors associated with prescription of opioids for joint pain	Medication error	Prospective cohort study	Gender: female gender is associated with decreased frequency of opioid prescription. Ethnicity: not studied. Income: not studied. Education: not studied.	Good
15	Fleming-Dutra et al. (2014) [10]	USA	Racial disparities in diagnosis and antibiotic prescription for otitis media	Diagnostic error/ Medication error	Retrospective cohort study	Gender: not studied. Ethnicity: Black children are more likely to receive narrow-spectrum antibiotics for otitis media compared with non-black children who are more likely to receive broad-spectrum antibiotics; black children are 30% less likely than non-black children to be diagnosed with otitis media during ambulatory care visits. Income: not studied. Education: not studied.	Good

## Results

### Quality of included studies

The results of the quality assessment can be consulted in Table 2. Seven studies [8–14] were rated “good” while the other eight [15–22] were rated as “fair”.

### General description of the studies

Five of the fifteen studies are carried out in Europe [11, 12, 14–16] while ten are carried out in the United States [8–10, 13, 17–22]. Ten [8, 10, 12–16, 19, 20, 22] of the fifteen studies explicitly looked at social disparities in their research questions whilst the other five studies look at general factors associated with the occurrence of patient safety events.

### Equity in patient safety

The following results are clustered into the domains of the WHO-framework on patient safety: administrative errors, diagnostic errors, medication errors, and transition of care errors.

### Patient safety domains – Definitions

- ▪ Administrative error: failures to carry out a planned action or undertaking an incorrect action as part of the systems and processes involved in delivering care. This includes errors associated with records, tests and transitions of care, such as patient identification errors, poor information to the patient after discharge or inadequate follow-up of patients after diagnostic tests.
- ▪ Medication error: error in treatment prescribing, transcribing, dispensing, administration or monitoring; wrong medication, dose, frequency, administration route or patient.
- ▪ Diagnostic error: missed, delayed or wrong diagnosis.
- ▪ Transition of care errors: inappropriate transitions between home, hospital, residential care settings and consultations with different health care providers in out-patient facilities.

### Patient safety threats due to administrative procedures

Two of the fifteen studies [17, 21] report on administrative errors in primary care. The first study [17] finds that ethnic minorities have higher odds of experiencing harm and adverse consequences due to errors in the testing process (ordering, implementing, and performing the test, reporting results to the clinician, notifying the patient of the results and following up) compared to white patients. The second study [21] evaluates chronically ill patients’ perception about the coordination of care, and it describes no significant disparities regarding patients’ ethnicity and income. Gender and education differences are not described in neither of the two studies.

### Patient safety threats due to diagnostic procedures

Seven of the fifteen studies [9, 10, 12, 13, 16, 20, 22] describe social disparities in diagnostic procedures. Four of them [9, 12, 16, 22] describe gender disparities in diagnosis. Henning et al. [16] and Maserejian et al. [22] describe that women have a lower likelihood of receiving proper and timely diagnosis respectively of cancer and coronary heart disease, compared to men. Henning et al. [16] describes differences in the interpretation of clinical symptoms and referral patterns in patients with Urothelial carcinoma of the bladder (UCB) visiting the General Practitioner (GP) for primary consultation and demonstrates that, despite the fact that women have worse prognosis and there are no gender-related differences in clinical symptoms of UCB, they are more likely to be treated for alleged urinary tract infections without further referral to an urologist compared to men. Maserejian et al. [22] describes disparities in physicians’ diagnosis of coronary heart disease (CHD) using a factorial experiment presenting videotaped CHD symptoms, systematically altering patient gender, age, socioeconomic status (SES) and race, reporting that physicians are less confident about CHD diagnosis in middle-aged female patients, indicating that their gender and age combination misleads physicians. Contrarily, Hansen et al. [12] reports that men experience longer doctor delays, that is the timeframe from the first contact with the GP presenting with symptoms up to time of investigation. One study [9] reports no association between gender and diagnostic errors. Five [9, 10, 13, 20, 22] of the seven studies describe ethnic disparities in diagnosis. One study [20] describes that although black patients experience lower levels of depression than white patients, they are more likely to be underdiagnosed with depression during the first primary care visit compared to whites. Two studies [9, 22] find no association between ethnicity and diagnostic errors whilst Fleming-Dutra et al. [10] find the opposite. The latter study reports that black children are less likely to be diagnosed with otitis media, compared to their white counterparts despite presenting the same symptoms. One study [13] reports that diagnosis of diabetes mellitus type 2 by physicians is associated with race resulting in underdiagnoses for white patients despite the same symptoms as black and Hispanic patients, whilst the prevalence of undiagnosed signs and symptoms of diabetes in the community is patterned more strongly by income and education (SES) and not by ethnicity. Similarly, Eva et al. [9] reports that patients’ SES is not associated with the physicians’ change of opinion regarding their diagnosis.

### Patient safety threats due to medication and treatment procedures

Five of the fifteen studies [8, 10, 11, 14, 15] report social disparities in medication procedures. Three of the five studies describe gender disparities in medication procedures. A systematic review [14] describing gender differences in antibiotic prescription demonstrates that n the amount of antibiotics prescribed to women is 36% higher than that prescribed to men in the 16–34 years age group and 40% higher in the 35–54 years age group. In particular, the amount of cephalosporins and macrolides prescribed to women is 44 and 32% higher, respectively, than those prescribed to men. Additionally, Cooper et al. [15] reports that women have greater odds of receiving potentially inappropriate prescriptions while Green et al. [11] describes that women have lower likelihood of receiving opioid prescriptions for pain treatment. Two of the five studies [8, 10] describe ethnic disparities for black patients in medication procedures. Becker et al. [8] finds that blacks are less likely to receive opioids for pain treatment compared to whites. Lastly, Fleming-Dutra et al. [10] reports that among children with otitis media, white children are more likely to receive broad-spectrum antibiotics than their black counterparts. One [15] of the five studies describes income disparities, reporting a higher likelihood to receive potentially inappropriate prescriptions for low-income patients compared to their wealthier counterparts. Disparities based on the patients’ educational attainment are not described in neither of the five studies.

### Patient safety threats due to transition of care procedures

Two of the fifteen studies [12, 19] describe social disparities in the transition of care procedures. Both studies report an unequal referral pattern with regard to the patients’ gender, describing that women are more likely to be referred or undergo cardiac stress test compared to men [19] while Hansens et al. [12] reports that men are more likely to experience longer doctor-system delays, namely referral to the hospital, first in-hospital visit, referral to treatment and its initiation, compared to women. Only one [19] of the two studies describes ethnic disparities, reporting no association between ethnicity and transition of care procedures. One [12] of the two studies describes income disparities in regard to transition of care procedures, reporting that high-income women experience shorter system delays, but they longer doctor delays, compared to their less wealthy counterpart.

## Discussion

Attention to patient safety in healthcare has increased dramatically over the years. Nonetheless, it is ambiguous that most of the patient safety research has been concentrated in the hospital setting and not in primary care [23] despite the 85% of all healthcare contacts occur in primary care [24]. While gender and ethnic disparities are documented in the existing patient safety literature, disparities regarding income and educational attainment are studied to a lesser extent. This literature review describes social disparities in patient safety in the primary care setting. The findings of this review are quite heterogeneous, however, they suggest that some vulnerable social groups are more likely to experience adverse patient safety events.

Previous research has shown that women are not offered the same diagnostic and therapeutic treatment compared to men [25–28] and that blacks are disadvantaged in receiving several medical services and procedures compared to other ethnic groups [29–34]. Our results confirm that, in primary care, women and black patients are more likely to receive inappropriate diagnosis [10, 12, 13, 22], treatment [8, 10, 11, 14, 15], or referrals [16, 19] compared to men and Whites respectively. However, our findings interestingly suggest that social disparities in patient safety vary among social groups depending on the type of disease, treatment, or health service. Furthermore, it is important to mention that only a limited number of studies describe the association between socioeconomic status and patient safety events, indicating a gap in the existing literature.

Although the egalitarian principle of equity claims that people in equal need of care should be treated equally, [35, 36] this systematic review shows some examples of inappropriate care with patients presenting the same conditions, as a result of gender, ethnicity or socioeconomic disparities. High-quality and safe care should be equally achievable for all patients [36] and should not differ between social groups [2]. Nevertheless, this systematic review offers an additional view to patient safety events in primary care. Individual intrinsic characteristics such as genetic, biological and physiologic factors and not necessarily explicit physicians’ bias may play a significant role in generating these disparities, confirming our claim that safety incidents are more likely to occur among vulnerable patient groups depending on the type of disease, treatment or healthcare service. As a matter of fact, the differences in the anatomy and physiology of men and women [37], as well as the clinical symptoms [16], or race [38] can play a significant role in misdiagnosis. For instance, Metersky et al. [38] reports that Blacks are less likely to be detected with pressure ulcers because of darkly pigmented skin and a study [39] carried out in London reports that Bangladeshi patients are more likely to present non-classic symptoms of acute myocardial infarction pain compared to Whites, making the initial diagnosis more difficult.

This review offers detailed insights that could generate valuable discussion among GPs about possible causes and explanations for disparities in patient safety in the primary care. These differences could arise from doctors’ awareness and perceptions of differences in illness prevalence within a specific patient group; as well as from patients’ culture and assertiveness to demand inappropriate prescriptions and to undergo inappropriate testing. Furthermore, the way healthcare is organised may play a major role in generating these disparities, making the findings more difficult to generalise.

Finally, we believe that the available data on patient safety incidents may be underestimated. It should be noted that measuring the real entity and magnitude of patients safety can be complex given the fact that there could be a general tendency among healthcare professionals and patients in underreporting patient safety incidents because of possible repercussions [40–42].

### Strengths and limitations

The use of the framework based on the most recent WHO guidelines and the comprehensive approach of this study are certainly major strengths. This review of the literature has also some limitations. We used Pubmed/MEDLINE and Web of Science to search for relevant papers considering the topic of this review i.e. patient safety in primary care. However, an additional search in social science databases might have resulted in additional papers looking at the issue with a different theory base e.g. discrimination.

A considerable number of studies on patient safety refers to “adverse drug events” in randomised clinical trials in medication research. However, those have been excluded from the search and inclusion because these incidents may be attributable genetic, physiological factors of the individuals [38] rather than a dosage or prescription error. Furthermore, data on ethnic minorities other than Black/African- Americans such as Hispanics or Asians were not documented as often as in the hospital setting.

### Implications for future research

Data regarding social disparities in patient safety in primary care are somehow fragmented which do not allow to grasp a thorough overview of the problem. Future research should focus on matching data on patients’ gender, ethnicity and socioeconomic status, in countries where this type of data are collected, with the data of critical incident registers in order to observe whether these events differ according to patients’ characteristics and in which patient safety domain.

## Conclusions

Overall, the results of this systematic review suggest that social disparities in patient safety in primary care exist and that they vary across the different social groups depending on the type disease, treatment or healthcare service. Furthermore, they suggest that gender and ethnicity are frequently studied while education and income should draw the attention of future research. Finally, awareness on the factors influencing patient safety events in primary care can represent a valuable guidance to general practitioners in order to reduce their occurrence.

## Abbreviations

CASP: Critical Appraisal Skills Programme
CHD: Coronary Heart Disease
GP: General Practitioner
IOM: Institute of Medicine
SES: Socioeconomic Status
UCB: Urothelial Carcinoma Of The Bladder
WHO: World Health Organisation
